# Genome Sequence of *Staphylococcus pseudintermedius* Strain E140, an ST71 European-Associated Methicillin-Resistant Isolate

**DOI:** 10.1128/genomeA.00207-12

**Published:** 2013-03-07

**Authors:** Arshnee Moodley, Matthew C. Riley, Stephen A. Kania, Luca Guardabassi

**Affiliations:** a Department of Veterinary Disease Biology, Faculty of Health and Medical Sciences, University of Copenhagen, Frederiksberg C, Copenhagen, Denmark; b Department of Biomedical and Diagnostic Sciences, University of Tennessee, Knoxville, Tennessee, USA; c U.S. Army Medical Service Corps

## Abstract

We report the first genome sequence of the methicillin-resistant *Staphylococcus pseudintermedius* (MRSP) strain E140, isolated from a canine bite wound infection in Denmark. This strain represents the dominant clonal lineage associated with canine MRSP infections in Europe.

## GENOME ANNOUNCEMENT

*Staphylococcus pseudintermedius* is a commensal Gram-positive species residing on the skin and mucosae of dogs, and it is the most common bacterial pathogen associated with canine infections, predominantly skin infections. Methicillin-resistant *S. pseudintermedius* (MRSP) was first reported in Europe in 2006 (1). The prevalent clone circulating in Europe belongs to sequence type (ST) 71, which displays resistance to all antibiotics routinely used in small animal practice (1–3). This clonal type has also been reported in Canada and the United States, although less frequently (2). MRSP ST71 can be transmitted to veterinarians and dog owners (4, 5), and human infection has been reported as a result of exposure to a colonized dog (6).

The genome of an MRSP ST71 strain (E140) isolated from a canine bite wound infection in Denmark was sequenced using three platforms: (i) a Roche GS FLX sequencer generating 133,947 reads, with an average length of 569 bp; (ii) an Illumina paired-end (PE) technology (500-bp library) generating 300 Mb data and 3,333,334 paired reads; and (iii) an Ion Torrent 318 generating 3,752,077 reads with an average length of 190 bp. *De novo* genome assembly of the various read sets was performed using Geneious and DNAStar SeqMan NGen and produced (i) Roche 454 data, 86 contigs of ≥1,000 bp with a mean length of 34,063 bp; (ii) Illumina PE data, 26 contigs of ≥1,000 bp with a mean length of 106,456 bp; and (iii) Ion Torrent data, 73 contigs of ≥1,000 bp with a mean length of 94,909 bp. In addition, a high-resolution AflII whole-genome restriction map of E140 was generated using an Argus whole-genome mapping system (Opgen, Inc.). The whole-genome map was used to align contigs from different assemblies and to verify final genome assembly. Automated annotation of genes was performed on the RAST (Rapid Annotation using Subsystem Technology) server (7).

The draft genome of MRSP E140 has a size of 2,769,487 bp, which is ~150 to 200 kb larger than the two published methicillin susceptible *S. pseudintermedius* genomes of strain ED99 (8) and strain HKU10-03 (9). This shift in genome size is in part due to the presence of a novel ~30-kb prophage, as seen in the sequencing contigs and verified in the optical map. E140 has a GC content of 38.0% and has 2,678 coding sequences, 5 ribosomal operons, and 68 tRNAs. Methicillin resistance is attributed to the presence of the staphylococcal cassette chromosome *mec* element (SCC*mec*) type II-III associated with ST71 (2, 10).

The discovery of the genome sequence of this MRSP will facilitate future studies to understand the rapid spread, fitness, and pathogenesis of this multidrug-resistant, dominant clonal lineage, which causes infections in Europe and in North America. In addition, the genome sequence information will assist investigators in tracking the evolution of methicillin and multidrug resistance in this species as more MRSP genomes become available.

### Nucleotide sequence accession number.

The draft genome sequence of *S. pseudintermedius* E140 has been deposited in the GenBank database with the accession number ANOI01000001.

## References

[1] Loeffler A Linek M Moodley A Guardabassi L Sung JM Winkler M Weiss R Lloyd DH 2007 First report of multiresistant, mecA-positive *Staphylococcus intermedius* in Europe: 12 cases from a veterinary dermatology referral clinic in Germany. Vet. Dermatol. 18:412–421 17991158 10.1111/j.1365-3164.2007.00635.x

[2] Perreten V Kadlec K Schwarz S Grönlund Andersson U Finn M Greko C Moodley A Kania SA Frank LA Bemis DA Franco A Iurescia M Battisti A Duim B Wagenaar JA van Duijkeren E Weese JS Fitzgerald JR Rossano A Guardabassi L 2010 Clonal spread of methicillin-resistant *Staphylococcus pseudintermedius* in Europe and North America: an international multicentre study. J. Antimicrob. Chemother. 65:1145–1154 20348087 10.1093/jac/dkq078

[3] Vanni M Tognetti R Pretti C Crema F Soldani G Meucci V Intorre L 2009 Antimicrobial susceptibility of *Staphylococcus intermedius* and *Staphylococcus schleiferi* isolated from dogs. Res. Vet. Sci. 87:192–195 19268332 10.1016/j.rvsc.2009.01.011

[4] Paul NC Moodley A Ghibaudo G Guardabassi L 2011 Carriage of methicillin-resistant *Staphylococcus pseudintermedius* in small animal veterinarians: indirect evidence of zoonotic transmission. Zoonoses Public Health 58:533–539 21824350 10.1111/j.1863-2378.2011.01398.x

[5] van Duijkeren E Kamphuis M van der Mije IC Laarhoven LM Duim B Wagenaar JA Houwers DJ 2011 Transmission of methicillin-resistant *Staphylococcus pseudintermedius* between infected dogs and cats and contact pets, humans and the environment in households and veterinary clinics. Vet. Microbiol. 150:338–343 21420256 10.1016/j.vetmic.2011.02.012

[6] Stegmann R Burnens A Maranta CA Perreten V 2010 Human infection associated with methicillin-resistant *Staphylococcus pseudintermedius* ST71. J. Antimicrob. Chemother. 65:2047–2048 20601356 10.1093/jac/dkq241

[7] Aziz RK Bartels D Best AA DeJongh M Disz T Edwards RA Formsma K Gerdes S Glass EM Kubal M Meyer F Olsen GJ Olson R Osterman AL Overbeek RA McNeil LK Paarmann D Paczian T Parrello B Pusch GD Reich C Stevens R Vassieva O Vonstein V Wilke A Zagnitko O 2008 The RAST server: rapid annotations using subsystems technology. BMC Genomics 9:75 18261238 10.1186/1471-2164-9-75 PMC2265698

[8] Ben Zakour NL Bannoehr J van den Broek AH Thoday KL Fitzgerald JR 2011 Complete genome sequence of the canine pathogen *Staphylococcus pseudintermedius*. J. Bacteriol. 193:2363–2364 21398539 10.1128/JB.00137-11 PMC3133065

[9] Tse H Tsoi HW Leung SP Urquhart IJ Lau SK Woo PC Yuen KY 2011 Complete genome sequence of the veterinary pathogen Staphylococcus pseudintermedius strain HKU10-03, isolated in a case of canine pyoderma. J. Bacteriol. 193:1783–1784 21278300 10.1128/JB.00023-11 PMC3067675

[10] Descloux S Rossano A Perreten V 2008 Characterization of new staphylococcal cassette chromosome mec (SCCmec) and topoisomerase genes in fluoroquinolone- and methicillin-resistant Staphylococcus pseudintermedius. J. Clin. Microbiol. 46:1818–1823 18305127 10.1128/JCM.02255-07 PMC2395120

